# Transcriptomic profiling of rumen epithelium, liver, and muscle reveals tissue-specific gene expression patterns in Hu sheep

**DOI:** 10.1186/s12864-025-12311-4

**Published:** 2025-11-14

**Authors:** Xiaowei Jia, Jiaxiao Li, Yuanxin Zhang, Boya Tian, Shengyong Mao, Junhua Liu, Wenxi Qian

**Affiliations:** 1 College of Life Science and Technology, Tarim University, Alar, 843300 China; 2 Ruminant Nutrition and Feed Engineering Technology Research Center, College of Animal Science and Technology, Nanjing Agricultural University, Nanjing, 210095 China; 3 Laboratory of Gastrointestinal Microbiology, National Center for International Research on Animal Gut Nutrition, Nanjing Agricultural University, Nanjing, 210095 China

**Keywords:** Hu sheep, Rumen epithelium, Transcriptomics, Metabolic tissues, WGCNA, Growth performance

## Abstract

**Background:**

The rumen epithelium, liver, and muscle tissues are key metabolic organs in ruminants, each performing distinct yet interconnected roles in energy metabolism and growth. However, how these tissues coordinate their gene expression to meet metabolic demands in Hu sheep remains poorly understood.

**Results:**

RNA sequencing of 48 tissue samples (rumen epithelium, liver, and muscle tissues from 16 male Hu sheep) identified 30,171 transcripts, including 7,403 commonly expressed and 3,414 uniquely expressed genes. The rumen epithelium displayed the highest number of uniquely expressed genes but lower functional enrichment compared to the liver and muscle, emphasizing its specialized yet limited metabolic pathways. Functional enrichment analysis showed that the rumen epithelium was enriched in pathways related to epithelial renewal. Differential gene expression analysis of commonly expressed genes further distinguished these tissues, reinforcing the metabolic specialization of the rumen epithelium. Weighted Gene Co-expression Network Analysis (WGCNA) revealed distinct tissue-specific modules associated with tissues. Key hub genes from different co-expression modules included histone deacetylase 1 (*HDAC1*, MEblue module, rumen epithelium), alpha-1-microglobulin/bikunin precursor (*AMBP*, MEdarkslateblue module, liver), and SWI/SNF related, matrix associated, actin dependent regulator of chromatin subfamily d member 3 (*SMARCD3*, MEbrown module, muscle), which regulate epithelial renewal, metabolic detoxification, and growth, respectively. Modules correlated with dry matter intake (DMI) were mainly found in the rumen epithelium and liver, while average daily gain (ADG)-related modules were enriched in the liver and muscle, indicating tissue-dependent regulatory mechanisms linking metabolic networks to performance.

**Conclusions:**

This study reveals transcriptional specialization and inter-tissue coordination in Hu sheep metabolic organs, identifies tissue-specific gene modules associated with DMI and ADG, and highlights hub genes as potential targets for precision breeding and feeding strategies to improve feed efficiency and growth in Hu sheep.

**Supplementary Information:**

The online version contains supplementary material available at 10.1186/s12864-025-12311-4.

## Background

The rumen epithelium is a hallmark of ruminant evolution and a key adapted part that facilitates the efficient utilization of fibrous plant material [1, 2]. Unlike the simple glandular stomachs of monogastric animals, the rumen epithelium features a stratified squamous structure with keratinized layers, uniquely suited to withstand the abrasive effects of content and the microbial ecosystem [3–5]. This tissue plays a central role in ruminant physiology as the primary site for the absorption and metabolism of volatile fatty acids (VFAs), which serve as the main energy source for ruminants [6, 7]. Furthermore, the rumen epithelium has evolved unique adaptations to efficiently process microbial fermentation products, balance nutrient absorption, and maintain energy homeostasis [8–10]. Despite its pivotal role, the molecular mechanisms governing the metabolic specialization of the rumen epithelium are ignorant, particularly in comparison to other key metabolic tissues such as the liver and skeletal muscle.

The liver and skeletal muscle are equally vital for ruminant growth and metabolic efficiency, complementing the functions of the rumen epithelium [11, 12]. The liver acts as a metabolic hub, playing a critical role in nutrient homeostasis, detoxification, and energy production, consuming 18–25% of total body oxygen. Meanwhile, skeletal muscle, which constitutes over 50% of body mass, is the primary determinant of meat quality and a core site for energy metabolism [13, 14]. A profound comprehension of the metabolic profiles and the coordinated functional mechanisms within these distinct tissues is pivotal for optimizing livestock productivity [15–17]. However, how these tissues differentially regulate their transcriptomes to meet metabolic demands remains underexplored.

Hu sheep (*Ovis aries*), native to the Jiaxing and Taihu regions of Zhejiang Province, China, are a cornerstone of the domestic sheep industry due to their combination of high reproductive performance, rapid growth, and adaptability to intensive farming systems. Historically valued for fine-quality lambskin, Hu sheep have evolved into a dual-purpose breed with both reproductive and meat production advantages. In recent decades, driven by the increasing demand for mutton, Hu sheep have been widely adopted in large-scale meat sheep production, and have been successfully introduced to regions such as Xinjiang, Tibet, and Gansu [18–21]. Although previous studies have explored the metabolic pathways in Texel sheep, Texel x Scottish Blackface (TxBF), small-tail Han sheep, and Hu sheep (female), the direct economic value of Hu sheep as a meat breed—particularly in male individuals—has gained increasing attention. However, comprehensive transcriptomic analyses across the rumen epithelium, liver, and muscle in male Hu sheep remain limited [22–25]. Moreover, the connections of transcriptomic data with phenotypic traits, such as dry matter intake (DMI) and average daily gain (ADG), have not been systematically addressed in this breed.

To address this gap, we performed transcriptomic profiling of the rumen epithelium, liver, and muscle tissues in male Hu sheep. We first conducted functional enrichment analysis of uniquely expressed genes in each tissue, followed by differential expression analysis of the commonly expressed genes. Weighted Gene Co-expression Network Analysis (WGCNA) was then applied using tissue type as the phenotype to identify tissue-associated gene modules, and subsequently using DMI and ADG as phenotypes to explore gene–trait associations [26, 27]. Our findings reveal transcriptional specialization across tissues and provide a resource for genetic improvement of feed efficiency and growth.

## Methods

### Sample collection

This experiment was conducted in two separate trials under identical conditions, differing only in duration. The animals used in this study were sourced from a commercial farm in Huzhou, Zhejiang, China. Informed consent was obtained from the farm owner(s) for the use of animals in this study. The first trial took place from October 4, 2022, to January 12, 2023, while the second trial spanned from December 22, 2022, to April 1, 2023, at the same farm in Huzhou, Zhejiang, China. All animal procedures were approved by the Animal Care and Use Committee of Nanjing Agricultural University (NJAU. No20220903N09) and adhered to institutional guidelines for animal research. Sixteen male Hu sheep (initial body weight: 24.56 ± 0.97 kg), all with detailed genealogical records and in good health, were randomly selected from a fattening flock. Detailed information on the sampling distribution for each trial is provided in Table S1 (Additional file 1: Table S1). The study lasted 100 days, including a 10-day acclimation period. Each sheep was housed individually in an indoor slotted-floor enclosure (200 cm × 60 cm) under identical environmental conditions. Diets were formulated according to the national feeding standards for meat sheep and goats (Ministry of Agriculture and Rural Affairs of China, 2004), with detailed composition provided in Table S2 (Additional file 1: Table S2). Sheep were fed twice daily (08:00 and 17:00) with ad libitum water access, maintaining a feed residual of 10–15%. At the end of the trial, final body weights (44.00 ± 1.36 kg) were recorded. Sheep were slaughtered three hours post-feeding, and tissue samples (rumen epithelium, liver, and muscle) were collected from all animals within 30 min post-slaughter. The sheep were humanely stunned and exsanguinated following ethical and regulatory guidelines, ensuring rapid unconsciousness induction and minimal animal stress. Samples were promptly snap-frozen in liquid nitrogen and stored at −80 °C for subsequent RNA extraction.

### RNA extraction, library construction, and sequencing

Approximately 100 mg of each frozen tissue sample (rumen epithelium, liver, and muscle) was ground into powder using a sterilized mortar and pestle. Total RNA was extracted using TRIzol Reagent (Life Technologies, CA, USA) following the manufacturer’s protocol. RNA quality and concentration were evaluated using a NanoDrop 2000 spectrophotometer (Thermo Fisher Scientific, Wilmington, DE, USA), and RNA integrity was assessed using an Agilent Bioanalyzer 2100 (Agilent Technologies, CA, USA).

High-quality RNA samples were used for library preparation. mRNA was enriched using oligo(dT)-coated magnetic beads supplied with the Hieff NGS™ mRNA Isolation Master Kit (Yeasen Biotechnology, Shanghai, China; Cat. No. 12603ES). First- and second-strand cDNA synthesis was performed, followed by end-repair, 3’-end adenylation, and adapter ligation. DNA fragments were purified using Hieff NGS™ DNA Selection Beads (Yeasen Biotechnology, Shanghai, China; Cat. No. 12601ES56, a high-performance alternative to AMPure XP magnetic beads). PCR-amplified indexed libraries were evaluated for quality using an Agilent Bioanalyzer 2100 (Agilent Technologies, CA, USA). Sequencing was performed on an Illumina platform, generating 150 bp paired-end reads.

### Reads mapping, assembly, and annotation

Raw sequencing reads (FASTQ format) were processed using in-house Perl scripts provided by Biomarker Technologies Co., Ltd (Beijing, China) to remove adapter-containing reads, reads with poly-N, and low-quality reads. The resulting clean reads were subsequently evaluated for quality metrics, including Q20 and Q30 scores, GC content, and sequence duplication rates, using FastQC v0.11.9 [28]. Reads were aligned to the *Ovis aries* reference genome (Oar_v3.1) using HISAT2 (v2.0.4) [29]. Transcript assembly and quantification were then performed using the Reference Annotation-Based Transcript (RABT) assembly method implemented in StringTie v2.2.1 [30], which reconstructs known transcripts and predicts novel transcripts and genes by comparing assembled sequences with the reference genome annotation. Gene counts were obtained as the number of sequencing reads mapped to each transcript, and expression levels were normalized as fragments per kilobase of transcript per million mapped reads (FPKM) [31].

### Gene expression patterns across tissues

Gene expression levels were categorized into ranges based on mean FPKM values. For each gene, the mean FPKM was calculated across at least 80% of the samples, and genes were classified into six expression ranges: < 0.1, 0.1–1.1, 1–10, 10–100, 100–1000, and 1000–10,000. The number of genes in each category was counted and visualized to illustrate gene expression distribution across tissues. Principal component analysis (PCA) was performed on the gene expression data, with feature vectors and principal component scores computed. The factoextra package (v1.0.7) was employed for visualization, highlighting the distribution of tissue samples and their principal component characteristics [32]. Logarithmic transformation and scaling were applied to normalize data. Heatmaps of gene expression matrices were generated using the complexHeatmap package (v2.18.0). To ensure reliable expression detection, for each tissue we retained only genes with FPKM > 1 in all biological replicates; these were defined as tissue-expressed genes [33]. Based on these tissue-expressed gene sets, genes present in all three tissues were classified as commonly expressed, whereas those present in exactly one tissue were considered uniquely expressed. For cross-tissue analyses, the intersection of the three tissue-expressed gene sets (i.e., genes consistently expressed across all tissues) was used for differential expression analysis, whereas their union (i.e., genes expressed in at least one tissue) served as the “cross-tissue union gene set” for WGCNA construction. Gene Ontology (GO) and Kyoto Encyclopedia of Genes and Genomes (KEGG) enrichment analyses were conducted using the enrichGO() and enrichKEGG() functions of the clusterProfiler package (v4.12.0) [34]. Both raw and FDR-adjusted *P*-values were computed, and pathways with *P* < 0.05 were considered for exploratory interpretation when no significant terms remained after correction.

### Detection of uniquely expressed and commonly expressed genes

Uniquely expressed genes and commonly expressed genes were analyzed using clusterProfiler for functional annotation and enrichment. For each tissue, significantly enriched KEGG Level 3 pathways of uniquely expressed genes were grouped by their corresponding Level 2 categories, and the proportion of each category was calculated relative to the total significant pathways. Categories were ranked in descending order, and those whose cumulative proportion exceeded 50% were defined as “dominant categories,” with all corresponding Level 3 pathways retained for further analysis. Protein interaction networks were generated using the STRING database and visualized in Cytoscape v3.10.1 [35]. To evaluate the relationship between network connectivity and expression level, the top 20 hub genes per tissue were identified according to node degree in the PPI network. Their FPKM values were log_2_-transformed and compared across tissues, with differences visualized using boxplots.

### Comparison of expression profiles among tissues

The ggtern package (v3.5.0) was used to compare relative expression ratios of commonly expressed genes among tissues. Differentially expressed genes (DEGs) were identified using the DESeq2 package (v1.44.0) based on pairwise comparisons between tissues [36]. Genes with absolute log_2_ Fold Change (log_2_FC) ≥ 1.5 and a false discovery rate (FDR)-adjusted *P*-value < 0.01 were considered significant DEGs [37]. To evaluate the potential influence of covariates on DEG identification, differential expression analyses were performed under three model settings: (M1) without covariates, (M2) including body weight as a covariate, and (M3) including both body weight and age as covariates. The overlap ratios of DEGs identified by these models were calculated for each tissue pair comparison. Identified DEGs were subjected to KEGG pathway enrichment analysis using the KEGGREST package (v1.44.1), and the up- and down-regulated genes in each pathway were quantified. A pathway was defined as significantly up-regulated or down-regulated in a tissue if over 80% of its constituent genes showed higher or lower expression in that tissue compared to others.

### Cross-tissue functional and co-expressed gene network analysis

For cross-tissue co-expression analysis, the cross-tissue union gene set was used as the input dataset. Expression variability across tissues was quantified using the matrixStats package (v1.3.0) to calculate the median absolute deviation (MAD), and genes with MAD > 1.5 were retained, resulting in a filtered set of 8,733 genes for WGCNA construction. WGCNA was performed using the WGCNA package (v1.72.5) [26], with a soft threshold of 22 selected to ensure a scale-free topology. Gene modules were identified based on the topological overlap matrix (TOM) using dynamic tree cutting, with a minimum module size of 30. Module eigengenes were calculated and correlated with tissue traits to identify tissue-specific modules. For each module, we filtered genes with TOM values greater than 0.25 to reduce network complexity while retaining biologically meaningful interactions. The resulting networks were then exported for visualization in Cytoscape. Network statistics (e.g., degree, clustering coefficient) were computed using Cytoscape’s Analyze Network tool to quantitatively assess the topology of hub gene networks across tissues.

To investigate the relationship between tissue transcriptomes and phenotypic traits (DMI and ADG), WGCNA was conducted separately for rumen epithelium, liver, and muscle transcriptomes. Gene expression data (FPKM > 1) for each tissue were used to construct a signed weighted co-expression network using the WGCNA package (v 1.72.5) in R. Modules were identified using hierarchical clustering of the topological overlap matrix, with a minimum module size of 30 and a dynamic tree-cutting method. TBtools-II was used to perform upset plot drawing of different modules and differential gene analysis [38]. For each tissue, WGCNA modules significantly associated with a given trait (ADG or DMI) were summarized by module eigengenes (MEs), defined as the first principal component (PC1) of the expression profiles of all genes in the module. Cross-tissue correlations were then assessed between trait-associated modules in different tissues (e.g., rumen epithelium–liver for DMI; liver–muscle for ADG) across the same animals using Pearson’s correlation; Spearman’s rank correlation was additionally reported as a robustness check. *P*-values were adjusted by the Benjamini–Hochberg method.

### Statistical analysis

The numbers of expressed genes detected in the rumen epithelium, liver, and muscle were compared using a one-way ANOVA, followed by Tukey’s post-hoc test.

Differences in gene expression levels among tissues were analyzed using log₂-transformed FPKM values in a one-way ANOVA model (SPSS version 25.0, SPSS Inc.), with tissue type as the main factor. Tukey’s multiple range test was used to detect pairwise differences between means. All results are presented as the mean ± standard error of the mean (SEM). A *P*-value < 0.05 was considered statistically significant.

## Results

### Overview of transcriptomes

The quality and integrity of all 48 RNA samples were verified before sequencing (Additional file 1: Table S3). Subsequently, sequencing of rumen epithelium, liver, and muscle tissues yielded a total of 1,036,660,245 clean reads, corresponding to 309,803,751,842 clean bases (Additional file 1: Table S4). The average clean read count was approximately 21.51 million, 21.79 million, and 21.50 million for rumen epithelium, liver, and muscle tissues, respectively. Mapping these reads to the sheep genome (Oar_v3.1) achieved rates of 89.98% ± 1.78% for rumen epithelium, 92.07% ± 0.64% for liver, and 91.39% ± 0.71% for muscle (Additional file 1: Table S5). Across the three tissues, 30,171 transcripts were identified, including 26,423 in the rumen epithelium, 25,494 in the liver, and 25,131 in the muscle. The types of expressed genes varied significantly between tissues (Additional file 1: Table S6). Gene expression levels and the number of expressed genes per tissue were visualized (Additional file 2: Fig. S1a). Principal component analysis (PCA) revealed clear separation among the rumen epithelium, liver, and muscle samples, indicating distinct gene expression patterns across tissues (Additional file 2: Fig. S1b). Notably, a small subset of genes dominated transcript abundance: 127, 113, and 48 genes in the rumen epithelium, liver, and muscle, respectively, accounted for approximately 50% of total aligned reads (Additional file 2: Fig. S1c and Additional file 1: Table S7-9).

The top 10 genes with the highest abundance in the rumen epithelium were cytochrome c oxidase subunit 1 (*COX1*), cytochrome b (*CYTB*), carbonic anhydrase 1 (*CA1*), cytochrome c oxidase subunit 3 (*COX3*), ribosomal protein S11 (*RPS11*), ribosomal protein S2 (*RPS2*), ATP synthase F0 subunit 6 (*ATP6*), keratin 17 (*KRT17*), ribosomal protein S8 (*RPS8*), and ATP synthase F0 subunit 8 (*ATP8*); in the liver, they were albumin (*ALB*), alpha-2-HS-glycoprotein (*AHSG*), *COX1*, haptoglobin (*LOC101102413*), *ATP6*, apolipoprotein A2 (*APOA2*), retinol-binding protein 4 (*RBP4*), *CYTB*, alpha-1-microglobulin/bikunin precursor (*AMBP*), and transferrin (*TF*); and in the muscle, they were actin alpha 1 skeletal muscle (*ACTA1*), creatine kinase M-type (*CKM*), myosin light chain 11 (*MYL11*), glyceraldehyde-3-phosphate dehydrogenase (*GAPDH*), tropomyosin 1 (*TPM1*), troponin C2 fast skeletal type (*TNNC2*), translationally-controlled tumor protein (*TPT1*), tropomyosin 2 (*TPM2*), myoglobin (*MB*), and troponin I2 fast skeletal type (*TNNI2*) (Additional file 1: Table S10).

**Fig. 1:**
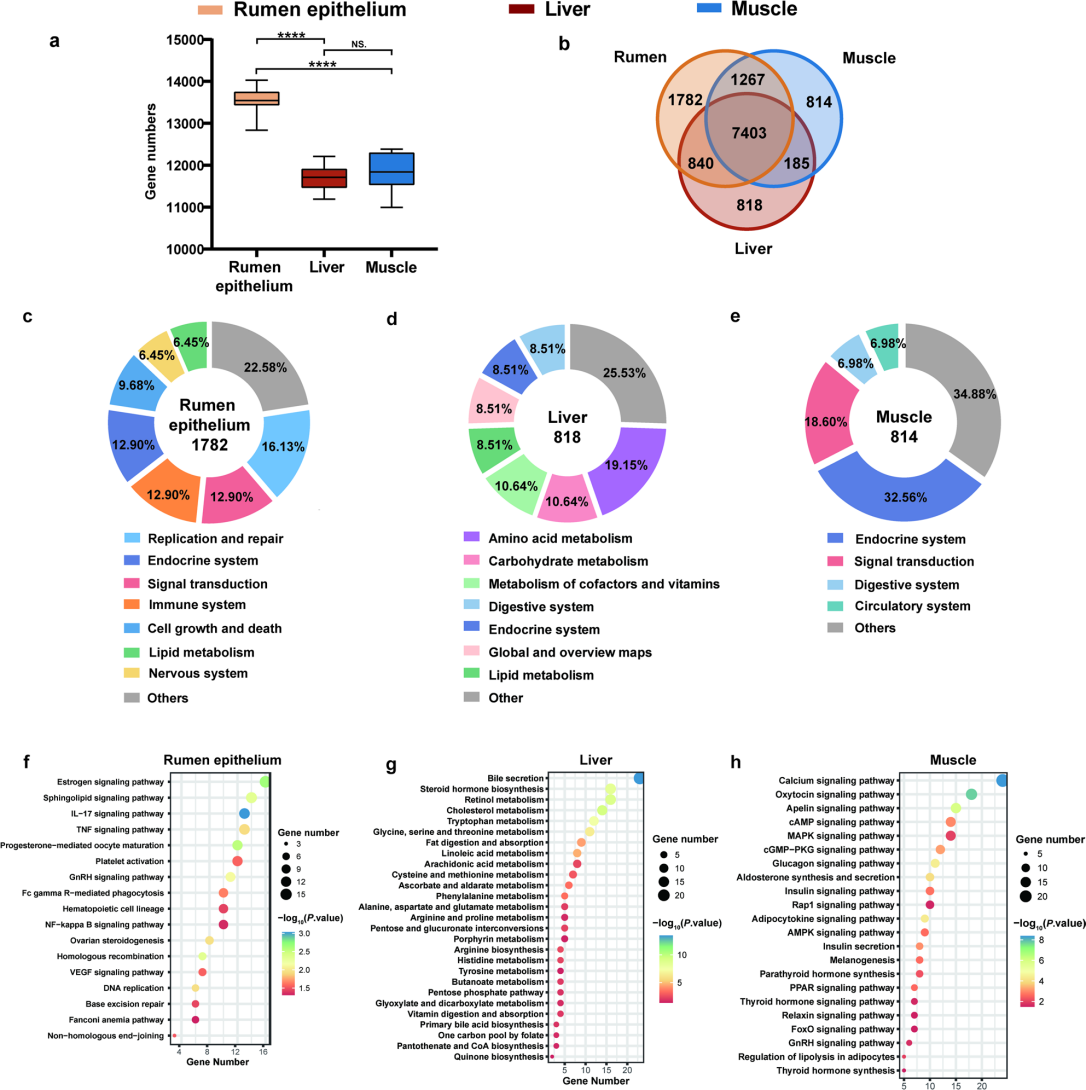
Expressed genes and functional categories in three tissues. **a** Numbers of expressed genes in the rumen epithelium, liver, and muscle. Statistical significance was determined using one-way ANOVA followed by Tukey’s post-hoc test (****P < 0.0001; NS., not significant). **b** Venn diagram of shared and unique expressed genes across tissues. **c-e** Proportion of KEGG Level 2 categories among uniquely expressed genes in each tissue. **f-h** KEGG Level 3 pathways within dominant Level 2 categories (cumulative proportion > 50% per tissue)

The rumen epithelium showed a significantly higher number of expressed genes (13,536 ± 310) compared to the liver (11,688 ± 316) and muscle (11,857 ± 420), with this difference being statistically significant (*P* < 0.0001). In contrast, no significant difference in gene expression was noted between liver and muscle (*P* = 0.370) (Fig. 1a). We identified 7,403 genes commonly expressed across all tissues. Unique expression profiles included 1,782, 818, and 814 uniquely expressed genes in the rumen epithelium, liver, and muscle, respectively (Fig. 1b). We observed that PCA based on the shared genes between the Hu sheep tissues agreed with the result of the PCA analysis in all transcripts (Additional file 2: Fig. S1d). GOBP secondary classification revealed that genes from the rumen epithelium were more enriched in categories annotated as “reproductive processes,” which primarily reflect cell cycle regulation, DNA repair, and epithelial renewal, rather than reproductive physiology, compared with the liver and muscle (Additional file 2: Fig. S2a). Moreover, GOCC secondary classification indicated that muscle exhibited more transcriptional regulatory activity and molecular carrier activity, rumen epithelium showed more cargo receptor activity and cytoskeletal movement, while liver displayed dominance in the remaining molecular functions (Additional file 2: Fig. S2b). The results of the KEGG secondary classification indicated that, when compared to the rumen epithelium and muscle, the liver exhibited a significantly reduced number of pathways related to signal transduction, the endocrine system, and replication and repair. Conversely, the liver demonstrated an increased number of pathways associated with lipid metabolism, amino acid metabolism, and the metabolism of cofactors and vitamins (Additional file 2: Fig. S2c).

### Functional analysis of uniquely expressed genes

KEGG pathway analysis highlighted differential enrichment across tissues, revealing 31, 47, and 43 significantly enriched pathways in the rumen epithelium, liver, and muscle, respectively (*P* < 0.05 and Additional file 1: Table S11-13). The rumen epithelium showed the highest proportion of pathways in replication and repair (16.13%), the liver was enriched in amino acid metabolism (19.15%), and the muscle was predominantly enriched in endocrine system pathways (32.56%) (Fig. 1c and e). Furthermore, 6, 31, and 25 KEGG pathways were highly enriched in the rumen epithelium, liver, and muscle, respectively (*P* < 0.01, Fold Enrichment (FE) > 2.5). The most enriched pathways were oas03440 (homologous recombination) in the rumen epithelium (*P* = 0.004, FE = 3.30), oas04610 (complement and coagulation cascades) in the liver (*P* < 0.001, FE = 9.78), and oas04820 (cytoskeleton in muscle cells) in muscle (*P* < 0.001, FE = 10.13). To further resolve functional specialization, significantly enriched Level 3 pathways were grouped by their corresponding Level 2 categories, ranked by proportion, and those whose cumulative proportion first exceeded 50% were defined as dominant categories (Fig. 1f-h). In the rumen epithelium, pathways such as estrogen signaling and platelet activation mediated by prostaglandins contained many genes, suggesting their potential importance in the functional maintenance of the rumen lining. In the liver, metabolic-related genes were found to be abundant, possibly reflecting the liver’s key role in metabolic control. The analysis for muscle tissue showed that pathways regulating actin cytoskeleton and muscle contraction were heavily populated, which correlates closely with the primary functions of muscle tissues.

**Fig. 2:**
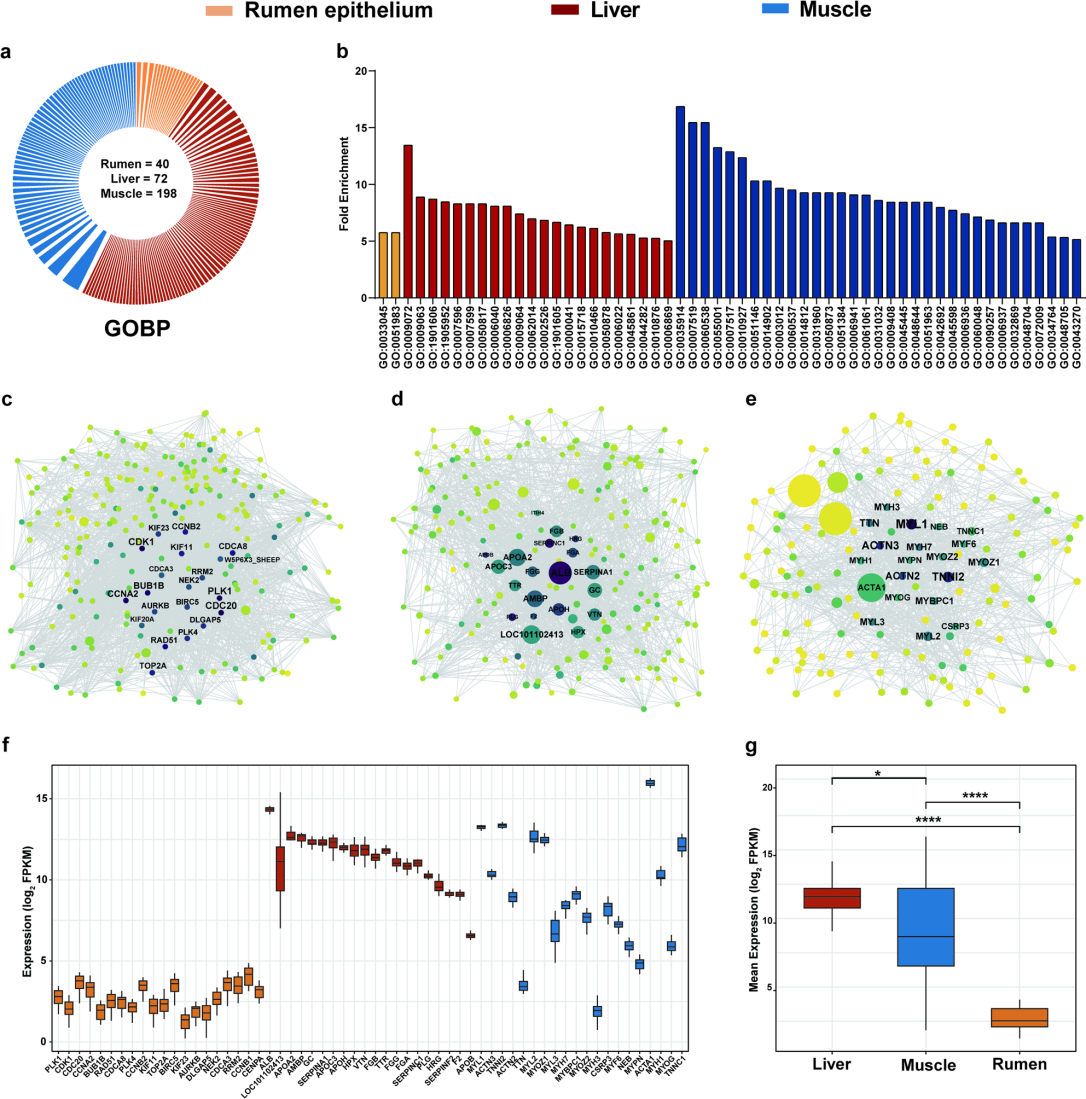
Enrichment and co-expression networks of uniquely expressed genes in rumen epithelium, liver, and muscle. **a** Pie chart displayed the number of significantly enriched GOBP terms in rumen epithelium (40), liver (72), and muscle (198), respectively (*P* < 0.05). Sector width represents the Fold Enrichment (FE) of GOBP terms. **b** Numbers of highly enriched GOBP terms in rumen epithelium (2), liver (22), and muscle (33) (*P* < 0.01, FE > 5). **c-e** STRING protein–protein association networks of uniquely expressed genes in (c) rumen epithelium, (d) liver, and (e) muscle. Nodes = genes; size = expression (FPKM); color = connectivity. Top 20 highest-degree genes are labeled. **f** Boxplots of log_2_(FPKM) values for the top 20 hub genes (ranked by connectivity) in each tissue across all three tissues. **g** Comparison of hub gene expression levels among tissues, grouped by tissue origin

Functional enrichment of uniquely expressed genes identified 93, 118, and 268 significantly enriched GO terms in the rumen epithelium, liver, and muscle, respectively, with 40, 72, and 198 GOBP terms enriched in these tissues (*P* < 0.05, Fig. 2a). Notable shared GOBP terms between the rumen epithelium and muscle included negative regulation of kinase activity (GO:0033673) and cellular response to stimulus (GO:0051716) (Additional file 1: Table S14-16). Notably, 2, 22, and 33 GOBP terms were highly enriched in the rumen epithelium, liver, and muscle, respectively (*P* < 0.01, FE > 5) (Fig. 2b). The top enriched GOBP terms were GO:0051983 (regulation of chromosome segregation) in the rumen epithelium (*P* = 0.003, FE = 5.78), GO:0009072 (aromatic amino acid metabolic process) in the liver (*P* < 0.001, FE = 13.47), and GO:0035914 (skeletal muscle cell differentiation) in the muscle (*P* < 0.001, FE = 13.47).

Analysis of gene connectivity, utilizing the STRING database, showed the highest connectivity in the rumen epithelium (up to 83), in contrast to 53 in the liver and 56 in muscle (Fig. 2c–e). Direct comparison of FPKM values confirmed that genes with high intramodular connectivity in the rumen epithelium network exhibited markedly lower expression levels than the top hub genes identified in the liver and muscle networks (Fig. 2f–g). This trend, initially observed through network visualization, was validated using log-transformed expression data, providing a more robust and quantitative assessment. The gene network in the rumen epithelium demonstrated greater complexity and heterogeneity, indicative of its involvement in diverse metabolic and regulatory processes. In contrast, the liver network was more compact and centralized, reflecting core functional gene dominance, while the muscle network retained heterogeneity and modularity, suggesting that specific physiological functions are driven by a subset of key genes.

### Function analysis and expression profile comparison among tissues of commonly expressed genes

**Fig. 3:**
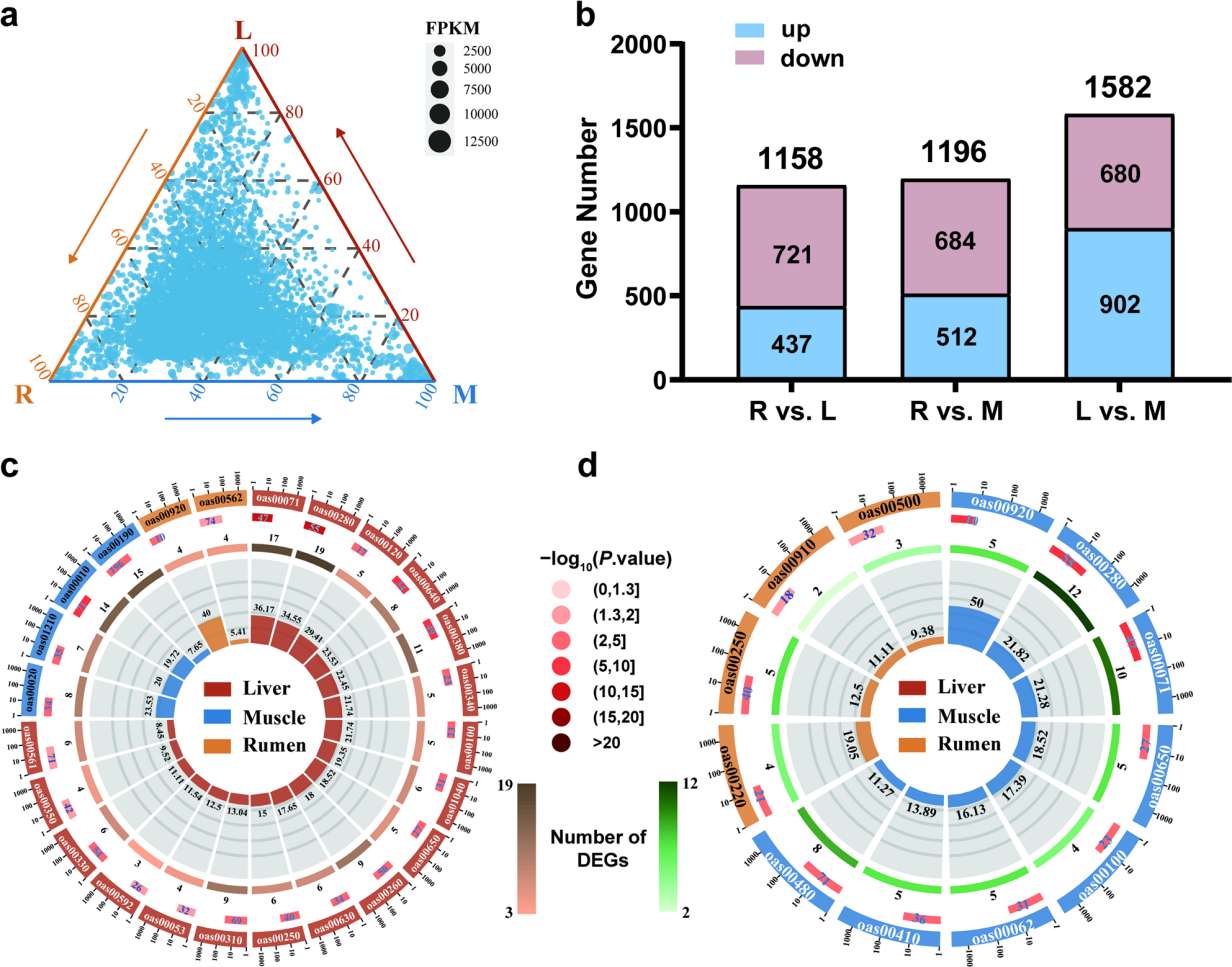
Differential expression and KEGG enrichment in three tissues. **a** Ternary plot of gene expression (FPKM) in rumen epithelium (R), liver (L), and muscle (M). Dot size = FPKM; position = relative proportion. **b** DEGs in pairwise comparisons (R vs. L, R vs. M, L vs. M). **c-d** Circular representation of KEGG pathway enrichment for up- (**c**) and down-regulated (**d**) Circular KEGG enrichment of up- (**c**) and down-regulated (**d**) DEGs. Outer ring = pathway IDs; 2nd ring = total genes (color = –log10 *P*); 3rd ring = DEG counts (color gradient = count); inner bars = Rich Factor × 100. Full pathway names and statistical details corresponding to KEGG IDs are provided in Tables S24–S26

To further investigate tissue-specific gene expression patterns, a ternary plot was employed to visualize the relative proportions of gene expression for 7,403 commonly expressed genes across the rumen epithelium (R), liver (L), and muscle (M). The plot revealed distinct clustering of genes near the vertex corresponding to the rumen epithelium, indicating higher expression levels of these genes in the rumen epithelium compared to both the liver and muscle (Fig. 3a and Additional file 1: Table S17-18). Functional enrichment analysis of these genes highlighted 340 GOBP, 156 GOCC, and 133 GOMF terms significantly enriched (*P* < 0.05), along with 104 enriched KEGG pathways at level 3 (Additional file 1: Table S19-22). At KEGG level 1, the predominant functional categories included metabolism (34.6%), genetic information processing (19.2%), organismal systems (17.3%), cellular processes (16.3%), and environmental information processing (9.6%).

To assess potential confounding effects of body weight and age, DEG analyses were repeated using models with these factors as covariates (M2: body weight; M3: body weight and age). The results showed > 99% overlap in DEGs among the three models under both significance thresholds, indicating negligible impact on downstream analyses (Additional file 1: Table S23).

Differential expression analysis further delineated tissue-specific functional adaptations. Significant DEGs included 1,158 between the rumen epithelium and liver (437 upregulated, 721 downregulated), 1,196 between the rumen epithelium and muscle (512 upregulated, 684 downregulated), and 1,582 between the liver and muscle (902 upregulated, 680 downregulated) (Fig. 3b). KEGG pathway analysis of DEGs revealed notable differences in metabolic activity among tissues. The liver exhibited markedly higher activity in amino acid and lipid metabolism pathways compared with the rumen epithelium and muscle, reflecting its pivotal role in metabolic homeostasis and energy regulation (Fig. 3c and Additional file 1: Tables S24–S26). This pattern highlights the liver’s specialized capacity for amino acid and lipid processing, which underpins its function as a central metabolic hub. No significant downregulation of major metabolic pathways was observed in the liver compared with either the rumen epithelium or muscle (Fig. 3d).

### Co-expression network of cross-tissue union gene set

**Fig. 4:**
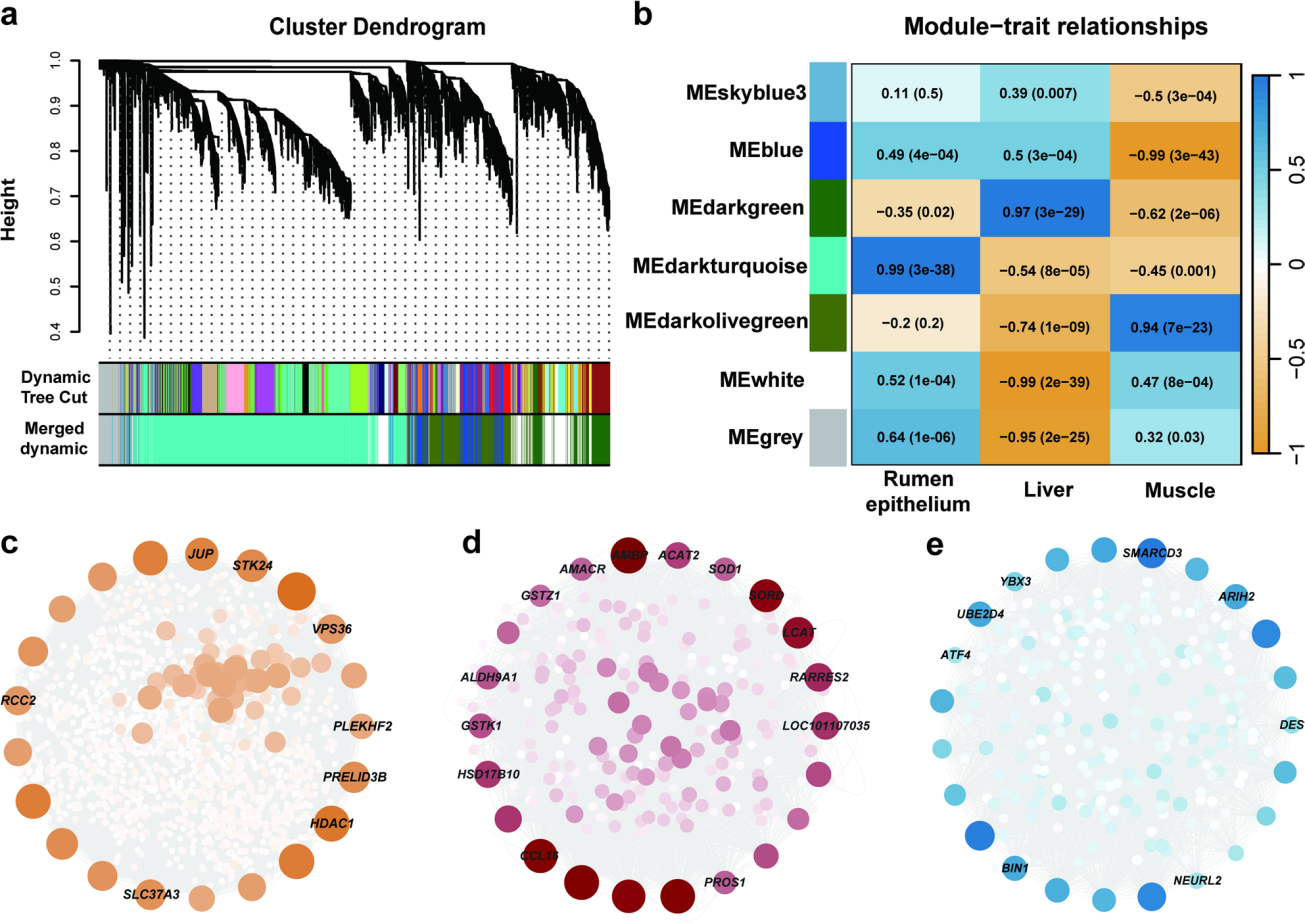
WGCNA reveals key hub genes associated with tissues. **a** Gene clustering dendrogram constructed from rumen epithelium, liver, and muscle samples. The upper color bar (“Dynamic Tree Cut”) shows initial modules identified by the dynamic tree cutting algorithm, whereas the lower color bar (“Merged Dynamic”) represents the final modules obtained after merging highly similar modules based on eigengene correlation. **b** Module–trait heatmap showing correlations (coefficients, *P*-values) between modules and tissues. Color scale represents Pearson correlation coefficients (blue = positive, orange = negative; intensity reflects correlation strength). **c-e** Hub-gene networks for (**c**) rumen epithelium, (**d**) liver, and (**e**) muscle. Top 20 genes by connectivity are highlighted; significant DEGs are marked. Node size and color intensity = degree of connectivity

To elucidate biological interactions and identify core driver genes across various tissues, we applied WGCNA to 8,733 genes, selected for their high variability (MAD > 1.5) and expression (FPKM > 1) from a total of 13,109 cross-tissue union gene set. A soft thresholding power (β) of 22 was applied, achieving a scale-free topology (R² = 0.85). Subsequently, the topological overlap matrix (TOM) was computed, followed by hierarchical clustering to group the genes into co-expression modules (Fig. 4a; Additional file 1: Table S27).

Our analysis delineated seven co-expression modules varying in size from 36 to 4,432 genes. Notably, the MEdarkgreen module, predominantly associated with liver tissue (*P* = 3 × 10⁻²⁹, *R* = 0.94), included 822 genes. The muscle-specific MEdarkolivegreen module contained 1,187 genes (*P* = 7 × 10⁻²³, *R* = 0.94), and the MEdarkturquoise module, closely linked to the rumen epithelium, comprised 4,432 genes (*P* = 3 × 10⁻³⁸, *R* = 0.99) (Fig. 4b). HUBGs were identified within each of these modules, with counts of 42, 60, and 222 in the MEdarkgreen, MEdarkolivegreen, and MEdarkturquoise modules, respectively. Several HUBGs identified in these modules were also uniquely expressed genes specific to their respective tissues, further emphasizing their tissue-specific regulatory roles. For instance, five genes (xylulokinase, *XYLB*; serpin family A member 1, *SERPINA1*; C-C motif chemokine ligand 16, *CCL16*; *AMBP*, and Coagulation factor VII, *F7*) were uniquely expressed in the liver’s darkgreen module, 19 genes (including cytochrome P450 26A1,*LOC101103439*; pleckstrin-2, *PLEK2*; G protein subunit alpha, *GNAI3*; transmembrane protein 45B isoform X1, *TMEM45B*; E3 ubiquitin-protein ligase CBL-C, *CBLC*; Krueppel-like factor 5, *KLF5* and others) were uniquely expressed in the rumen epithelium’s darkturquoise module. Additionally, four genes (dual specificity phosphatase 26, *DUSP26*; potassium inwardly rectifying channel subfamily J member 12, *KCNJ12*; neuralized E3 ubiquitin protein ligase 2, *NEURL2*; and Ribosomal protein L17, *LOC101109676*) were uniquely expressed in the muscle’s darkolivegreen module. Moreover, the top 20 hub genes are visualized in Cytoscape, highlighting their centrality within the co-expression networks of different tissues (Fig. 4c–e). To validate our observations, we quantitatively assessed the network structures of the rumen epithelium (MEdarkturquoise), liver (MEdarkgreen), and muscle (MEdarkolivegreen) modules using Cytoscape’s network analyzer. The MEdarkturquoise exhibited the highest node count (1,565), largest edge number (59,718), highest average degree (76.8), density (0.049), and centralization (0.830), indicating a highly interconnected and centralized structure. In contrast, the MEdarkgreen showed a moderately compact network (277 nodes, average degree 39.1), while the MEdarkolivegreen was characterized by lower density and higher modularity (257 nodes, density 0.101, heterogeneity 1.510), reflecting a more distributed and heterogeneous structure (Additional file 1: Table S28). These quantitative metrics corroborate the topological distinctions inferred from visual inspection.

### Weighted gene co-expression network analysis of DMI and ADG across rumen epithelium, liver, and muscle

**Fig. 5:**
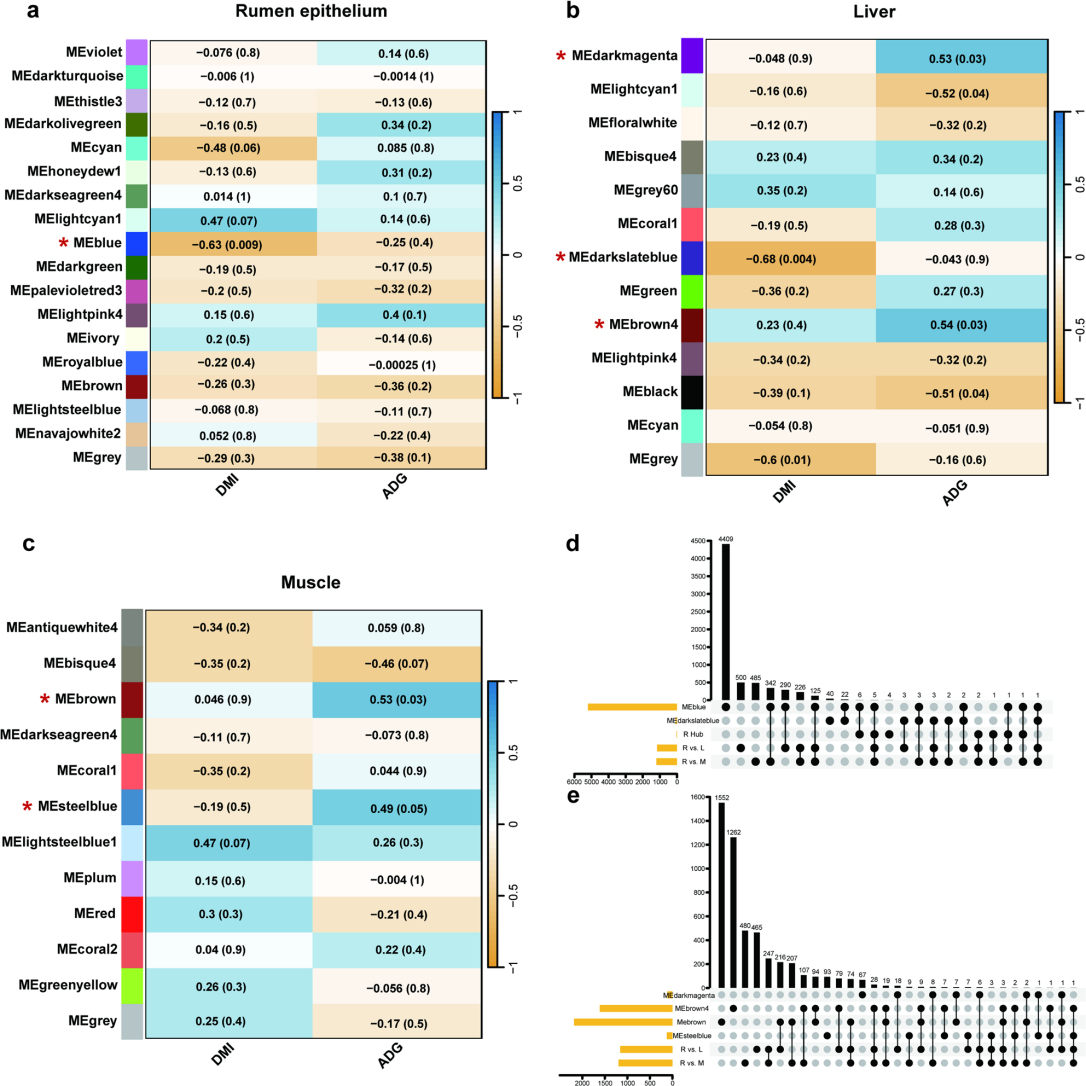
Module-trait relationships in rumen epithelium, liver, and muscle tissue. **a-c** Heatmaps of Pearson correlation coefficients between module eigengenes and DMI or ADG (*P*-values in parentheses). Color scale = Pearson correlation coefficients (blue = positive, orange = negative; intensity reflects correlation strength). **d** UpSet plot showing overlaps among DMI-negatively correlated modules (MEblue, MEdarkslateblue), R hub genes (from Fig. 4c), and DEGs from R vs. L and R vs. M in Fig. 3b. **e** UpSet plot showing overlaps among ADG-positively correlated modules (MEdarkmagenta, MEbrown4, MEbrown, MEsteelblue) and the same DEG sets. Y-axis = number of genes; yellow horizontal bars represent total genes per set, vertical black bars indicate intersection sizes, and numbers above bars denote gene counts

WGCNA identified 18, 13, and 12 gene modules in the rumen epithelium, liver, and muscle tissues, respectively, as illustrated in Fig. 5a-c (Additional file 1: Table S29). Among the identified modules, 8 exhibited significant associations with DMI and ADG (*P* < 0.05). In the rumen epithelium, the MEblue module was negatively correlated with DMI (*P* = 0.009, *R* = −0.63), indicating that genes within this module may be involved in ruminal adaptations to feed intake (Fig. 5a). In liver tissue, the MEdarkslateblue module demonstrated the strongest negative correlation with DMI (*P* = 0.004, *R* = −0.68), while four additional modules were significantly associated with ADG (*P* < 0.05) (Fig. 5b). In skeletal muscle, the MEbrown and MEsteelblue modules showed significant correlations with ADG (*P* < 0.05; Fig. 5c). The strongest module-trait relationships included the MEblue module-DMI relationship in rumen epithelium (*P* = 0.009, *R* = −0.63, 5207 genes) and the MEbrown4 module-ADG relationship in liver (*P* = 0.03, *R* = 0.54, 1612 genes) (Fig. 5a-b). 28 genes were shared between the MEblue in the rumen epithelium and the MEdarkslateblue in the liver, both negatively correlated with DMI (Fig. 5d). Within these intersecting modules, *JUN* (Jun proto-oncogene, AP-1 transcription factor subunit) was also differentially expressed across tissues, suggesting a potential cross-tissue transcriptional role in feed intake regulation. In parallel, *JUP* (intercellular junctions), *VPS36* (Vacuolar protein sorting 36 homolog), *STK24* (Serine/threonine kinase 24), *RCC2* (Regulator of chromosome condensation 2), and *SLC37A3* (Solute carrier family 37 member 3) were identified at the intersection of the MEblue module, R hub genes, and DEGs (R vs. L and R vs. M), highlighting their potential cross-tissue regulatory significance (Fig. 5d; Additional file 1: Table S30). Likewise, 147 genes were shared in the module-ADG relationship between liver and muscle tissues, with notable genes including cell death inducing p53 target 1 (*CDIP1*), nuclear receptor subfamily 1 group H member 3 (*NR1H3*), and methionine sulfoxide reductase B3 (*MSRB3*), which were DEGs across tissue pairwise comparisons (Fig. 5e; Additional file 1: Table S30). Interestingly, these genes exhibited lower expression levels in the rumen epithelium compared to the liver and muscle. For instance, in rumen vs. liver comparisons, *NR1H3*, *MSRB3*, and *CDIP1* showed log₂FC values of − 2.17 (*P* = 1.12 × 10⁻⁸⁸), − 5.07 (*P* < 1 × 10⁻³⁰⁰), and − 2.81 (*P* = 3.43 × 10⁻⁶⁷), respectively. Similar patterns were observed in rumen vs. muscle comparisons, with *NR1H3*,* MSRB3* and *CDIP1* markedly downregulated in the rumen epithelium (Additional file 1: Table S31). Motivated by the 28 genes commonly associated with the module–DMI relationship in these two tissues, Pearson’s analysis revealed a significant positive correlation between the rumen (MEblue) and liver (MEdarkslateblue) (*r* = 0.540, FDR = 0.0309). This supports coordinated transcriptional programs between digestive and metabolic tissues in relation to DMI (Additional file 2: Fig. S3a; Additional file 1: Table S32). Pearson’s analysis identified a strong positive correlation between the liver (MEdarkmagenta) and muscle (MEsteelblue) (*r* = 0.804, FDR = 7.07 × 10⁻⁴), whereas other liver–muscle pairs did not reach significance after FDR correction. Spearman’s analysis showed a similar but non-significant trend for the same pair (*r* = 0.462, FDR = 0.295) (Additional file 2: Fig. S3b; Additional file 1: Table S32).

## Discussion

In this study, we integrated transcriptome data from the rumen epithelium, liver, and muscle tissues of Hu sheep. Despite the distinct functional roles of these tissues, their transcriptomes are dominated by the expression of a relatively small subset of genes, consistent with findings in human and beef cattle transcriptomes [37]. Similar to the findings in beef cattle reported by Sun et al. (2019), we found that several highly expressed transcripts, such as *COX1* and *KRT17* in the rumen epithelium, *ALB*, *COX1*, and *TF* in the liver, and *ACTA1* and *TPM2* in muscle, were also present in Hu sheep [15]. This overlap highlights shared key functional elements across species and tissues [33]. Our analysis also revealed the unique gene expression dynamics in the rumen epithelium. Specifically, the rumen epithelium exhibited a significantly larger number of genes in both the very low (FPKM < 0.1) and high expression ranges (FPKM > 1000) compared to the liver and muscle [15, 39]. This suggests a more complex and diverse expression pattern in the rumen epithelium. Additionally, more genes in the rumen epithelium accounted for around 50% of total aligned reads, indicating that it relies on a relatively small number of highly expressed genes to maintain core physiological functions [39, 40]. In contrast, the liver and muscle exhibited more consistent expression levels, emphasizing the different regulatory strategies each tissue uses to maintain metabolic homeostasis. Mitochondrial genes *CYTB* and *COX3* stood out for their high and stable expression across all tissues, underscoring their critical roles in oxidative phosphorylation and energy maintenance [41, 42]. Their consistently high expression with low variation across the rumen epithelium, liver, and muscle indicates their key role in maintaining cellular respiration and energy homeostasis in metabolically active tissues. Like *COX1*, a stable housekeeping gene in various species, *CYTB* and *COX3* may serve as reliable reference genes for quantitative gene expression analysis in ruminants, providing a stable baseline for normalizing target gene expression across different physiological conditions [43]. The widespread expression of *CYTB* and *COX3* underscores their importance in supporting tissue-specific metabolic demands. These findings suggest their potential use in cross-tissue comparisons and livestock genetic studies, with further research needed to confirm their suitability as reference genes under varying physiological and environmental conditions.

Interestingly, while the rumen epithelium showed the highest number of uniquely expressed genes, these genes were associated with fewer GOBP terms and KEGG pathway enrichments than the liver and muscle. This is in line with previous findings in beef cattle, suggesting that the rumen epithelium, while functionally specialized, operates within a more constrained metabolic network [15]. Notably, GOBP analysis revealed that one of the most enriched terms in the rumen epithelium was the regulation of chromosome segregation (GO:0051983), highlighting the tissue’s role in maintaining cellular integrity and mitotic regulation [44–46]. The corresponding unique genes in this GO term were Zwilch kinetochore protein (*ZWILCH***)**, Cell division cycle 2 protein isoform 1 (*CDK1*), Spindly (*SPDL1*), and Mitotic checkpoint serine/threonine-protein kinase BUB1 beta (*BUB1B*), which have been reported to have multiple roles in epithelium cell-cycle regulation and chromosome separation in the sheep and human [46–48]. Moreover, the presence of genes like *CDK1* and *BUB1B* in the rumen epithelium, despite their low expression levels, underscores the importance of gene connectivity in regulating key processes like cell division and genome stability [49]. The rumen epithelium gene network showed lower expression levels for highly connected genes compared to those in the liver and muscle, indicating that gene connectivity rather than expression level alone plays a crucial role in regulating key biological processes. Additionally, the secondary classification of KEGG pathways further supported the specialized role of the rumen epithelium, with significant enrichment in pathways related to replication and repair (16.13%). These findings are consistent with the constant exposure of the rumen epithelium to microbial fermentation products and abrasive food particles, necessitating robust repair mechanisms [50, 51]. In comparison, the liver was enriched in pathways related to amino acid and lipid metabolism, reflecting its central role in nutrient processing and detoxification [52, 53]. Muscle tissue was predominantly enriched in endocrine signaling pathways, which are critical for regulating energy metabolism and growth, particularly concerning muscle development and maintenance [54].

When comparing the expression patterns of commonly expressed genes across the rumen epithelium, liver, and muscle, the rumen epithelium displayed distinct advantages in inositol phosphate and sulfur metabolism pathways. Specifically, the upregulation of genes like phospholipase C delta 1 (*PLCD1*), phospholipase C beta 3 (*PLCB3*), Inositol-3-phosphate synthase 1 (*ISYNA1*), and Inositol-trisphosphate 3-kinase C (*ITPKC*) linked to the Ca^2+^ signaling pathway underscores the critical role of calcium signaling in rumen epithelium development [55]. Sulfur metabolism, in particular, is integral to microbial fermentation and hydrogen sulfide metabolism within the rumen, highlighting the specialized function of the rumen epithelium in maintaining its microbial ecosystem [56]. Despite the rumen epithelium’s higher number of highly expressed genes, differential expression analysis revealed fewer upregulated genes [15]. This suggests that the rumen epithelium focuses on homeostatic regulation, maintaining consistent gene expression in its stable digestive environment, rather than frequently adjusting gene expression, as observed in the liver and muscle. In contrast, muscle tissue demonstrated distinct metabolic profiles, with advantages in glycolysis and oxidative phosphorylation, consistent with its role as the primary energy-consuming tissue in the body [57, 58].

Using WGCNA, we delineated the network structure and function of gene co-expression modules highly correlated with specific tissues. In the MEdarkturquoise module, the top 20 hub genes with DEGs were found to play essential roles in several key biological processes. These included metabolite transport (*SLC37A3*), intercellular junctions (*JUP*), signal transduction (Pleckstrin homology and FYVE domain containing 2, *PLEKHF2*), cytoskeleton and cell cycle regulation (*STK24*; *RCC2*), energy metabolism (PRELI domain containing 3B, *PRELID3B*), and protein degradation (*VPS36*) [59–63]. Interestingly, the specific roles of these genes in the rumen epithelium remain largely unexplored, as much of the existing research has focused on human studies. Notably, *HDAC1*’s prominent role in this module underscores its significance in chromatin remodeling and cellular turnover, critical for epithelial renewal and response to environmental stimuli [49]. In the MEdarkgreen module, the top 20 hub genes are central to liver function, with roles in metabolism (Acetyl-CoA acetyltransferase 2, *ACAT2*; aldehyde dehydrogenase 9 family member A1, *ALDH9A1*; alpha-methylacyl-CoA racemase, *AMACR*; hydroxysteroid 17-beta dehydrogenase 10, *HSD17B10*; lecithin-cholesterol acyltransferase, *LCAT*; carbonyl reductase [NADPH] 2, *LOC101107035*; retinoic acid receptor responder 2, *RARRES2*; sorbitol dehydrogenase, *SORD*), antioxidant activity (glutathione S-transferase kappa 1, *GSTK1*; glutathione S-transferase zeta 1, *GSTZ1*; superoxide dismutase 1, *SOD1*), and immune and inflammatory regulation (*CCL16*; protein S, *PROS1*) [64–68]. The high connectivity of *AMBP* within this module further emphasizes the liver’s function in protecting cells from oxidative stress and maintaining cellular health [69]. This is particularly vital for ruminants, as the fermentation process in their digestion produces many metabolic by-products that the liver must detoxify [70]. Finally, the MEolivegreen module associated with the muscle highlights genes involved in structural integrity (bridging integrator 1, *BIN1*; desmin, *DES*), protein degradation (ariadne RBR E3 ubiquitin protein ligase 2, *ARIH2*; ubiquitin conjugating enzyme E2 D4, *UBE2D4*; neuralized E3 ubiquitin protein ligase 2, *NEURL2*), and stress responses (activating transcription factor 4, *ATF4*; Y-box binding protein 3, *YBX3*) [71–76]. SWI/SNF related, matrix associated, actin dependent regulator of chromatin, subfamily d, member 3 (*SMARCD3*) exhibited the highest connectivity in this module. Previous studies have linked *SMARCD3* to eight quantitative trait loci (QTLs) associated with ADG, suggesting that *SMARCD3* regulates growth-related genes through chromatin remodeling, thereby influencing ADG and overall weight gain in livestock [77].

Moreover, we also conducted WGCNA to identify modules associated with animal performance (DMI and ADG) across the rumen epithelium, liver, and muscle. The MEblue module in the rumen epithelium and the MEdarkslateblue module in the liver were both significantly correlated with DMI, suggesting their potential roles in feed intake regulation. Conversely, ADG-related modules, such as MEdarkmagenta and MEbrown4 in the liver, along with MEbrown and MEsteelblue in the muscle, emphasize the tissue-specific contributions to growth performance, corroborating findings from previous studies that have indicated tissue-specific impacts on overall animal performance [15, 78]. Our study uncovered overlaps in gene modules associated with DMI and ADG across various tissues. The overlap of DMI-associated genes in these two tissues suggests a synchronized metabolic response to nutrient intake, which is crucial for optimizing the conversion of ingested feed into energy [10, 79]. Among these genes, *JUN* emerged as the only differentially expressed gene, implying coordinated transcriptional control between rumen and liver in DMI regulation. Additionally, *JUP*, *VPS36*, *STK24*, *RCC2*, and *SLC37A3* were identified as cross-tissue hub genes connecting the MEblue module and DEGs, reflecting their potential roles in epithelial integrity, vesicle trafficking, and signal transduction [62, 80]. Additionally, our analysis identified ADG-related modules in the liver and muscle, suggesting a shared regulatory framework that influences growth [81]. Prominent among these genes, *CDIP1*, *NR1H3*, and *MSRB3* were differentially expressed across the tissues. These genes play vital roles in lipid metabolism, inflammatory responses, and antioxidant defense, underscoring their importance in tissue-specific metabolic regulation [82–84]. Intriguingly, these genes exhibited lower expression levels in the rumen epithelium compared to the liver and muscle, pointing to a specialized metabolic focus of the rumen epithelium on fermentation and nutrient absorption, distinct from the broader metabolic functions observed in the liver and muscle. Consistent with this tissue specialization, our cross-tissue eigengene correlation analyses revealed coordinated transcriptional programs between the rumen epithelium and liver in relation to DMI, and between the liver and muscle in relation to ADG, providing direct network-level support for metabolic interactions among these tissues. These insights into the transcriptional regulation associated with key performance traits in Hu sheep highlight the coordinated roles of multiple tissues in determining feed efficiency. While modules such as MEblue and MEdarkslateblue were defined within the specific context of this dataset, their associations with DMI and tissue-level expression patterns provide valuable, dataset-driven hypotheses for understanding the molecular basis of nutrient utilization. Future research should validate these findings across independent populations and explore the causal mechanisms linking differential expression and module associations to improve breeding and nutritional strategies in ruminants, while integrative multi-omics and single-cell transcriptomic approaches will be crucial to further resolve the cellular mechanisms and intricate regulatory hierarchies governing these traits [85–88].

## Conclusion

In conclusion, our transcriptomic analysis of rumen epithelium, liver, and muscle in Hu sheep revealed distinct tissue-specific gene expression patterns and metabolic functions: the rumen epithelium was enriched in pathways for nutrient absorption and epithelial integrity, the liver in amino acid and lipid metabolism, and the muscle in endocrine regulation and structural maintenance. WGCNA identified modules strongly associated with performance traits. For DMI, the MEblue (rumen epithelium) and MEdarkslateblue (liver) modules were both negatively correlated, with *JUN* identified as the only cross-tissue DEG. For ADG, modules in the liver and muscle contained *CDIP1*, *NR1H3*, and *MSRB3*, all differentially expressed across tissues. These findings provide molecular targets for precision breeding and nutritional strategies to improve feed efficiency and growth in ruminants, while underscoring the coordinated yet specialized roles of different tissues in regulating performance traits.

## Supplementary Information

Additional file 1: Tables S1–S32.

Additional file 2: Figures S1–S3.

## Data Availability

The raw sequencing data generated in this study have been deposited in the NCBI Sequence Read Archive (SRA) under BioProject accession number PRJNA1226734. These data will be publicly available from February 22, 2025.

## Abbreviations

ADG: Average daily gain
DMI: Dry matter intake
COV: Coefficient of variation
DEGs: Differentially expressed genes
PCA: Principal component analysis
GO: Gene ontology
KEGG: Kyoto encyclopedia of genes and genomes
MAD: Median absolute deviation
TOM: Topological overlap matrix
WGCNA: Weighted gene co-expression network analysis
