# Supplementary material for: Transcriptomic profiling of rumen epithelium, liver, and muscle reveals tissue-specific gene expression patterns in Hu sheep
Source: BMC Genomics. 2025 Nov 14;26:1115. doi: 10.1186/s12864-025-12311-4 (PMC12729863; doi:10.1186/s12864-025-12311-4)
Supplement: Supplementary file 2 — Additional file 2: Figures S1–S3. [file 12864_2025_12311_MOESM2_ESM.docx]

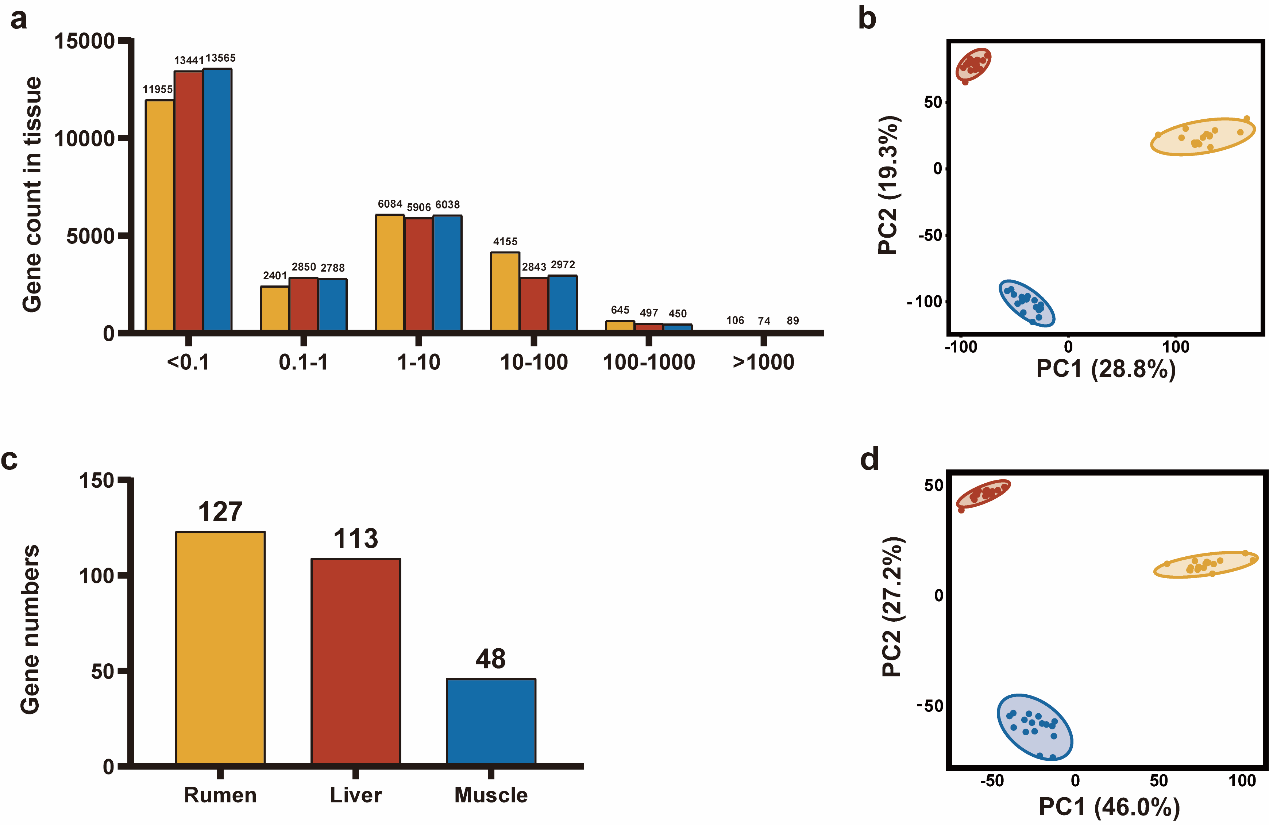


**Fig. S1** (**a**) Gene count in tissue: Displays the distribution of different gene expression levels across various tissues. (**b**) The PCA based on shared genes shows differences in gene expression between muscle, rumen epithelium, and liver. (**c**) Number of genes contributing to 50% of total expression (FPKM) in each tissue, calculated by ranking genes by mean FPKM and summing until 50% was reached. (**d**) The PCA based on shared genes shows gene expression differences among tissues. The bars in yellow, red, and blue represent the rumen epithelium, liver, and muscle tissues, respectively.


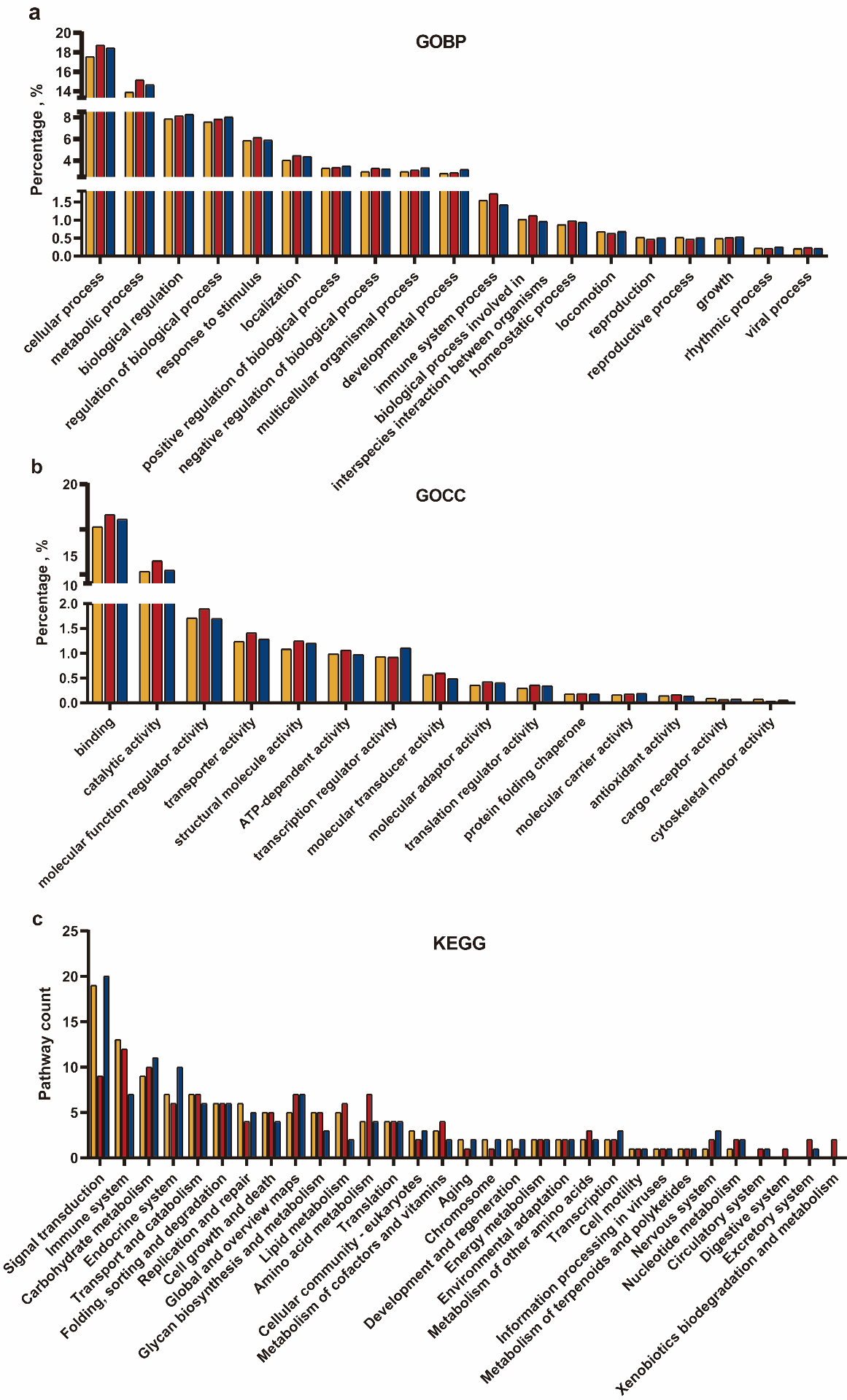


**Fig. S2** (**a**) GOBP classification, (**b**) GOCC classification, and (**c**) KEGG pathway enrichment across various tissues. The bars in yellow, red, and blue represent the rumen epithelium, liver, and muscle tissues, respectively.


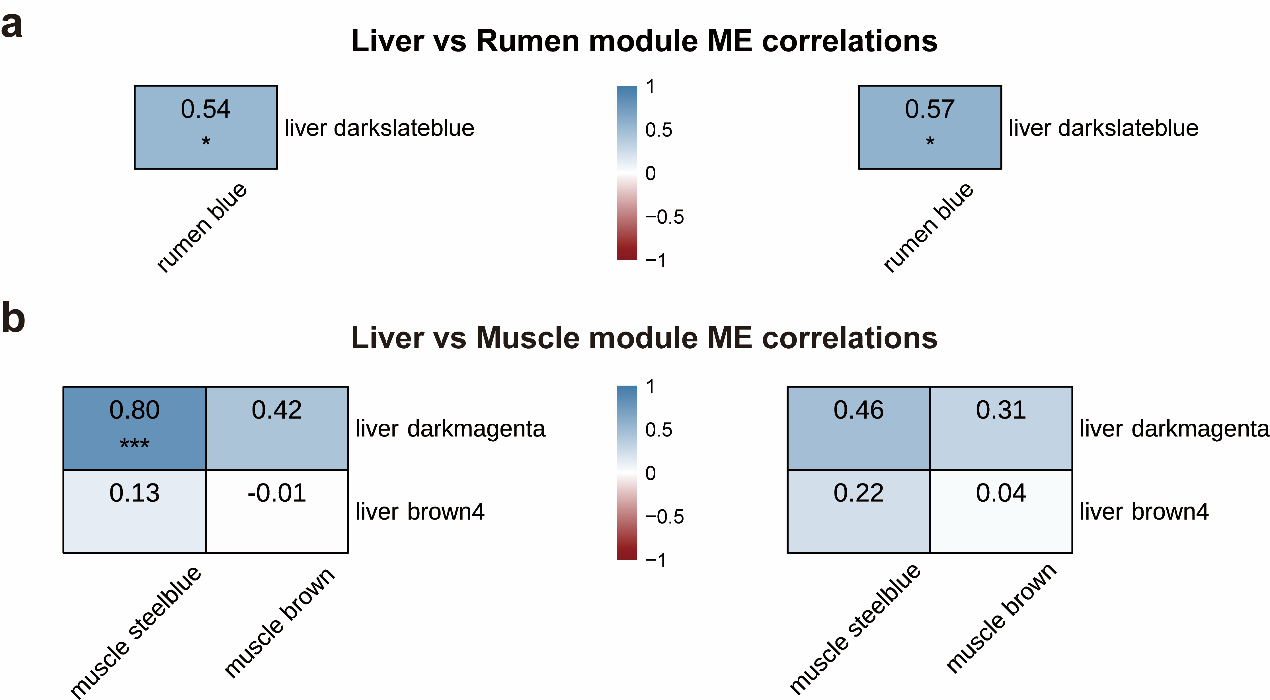


**Fig. S3** (**a**) Correlation heatmap between liver and rumen modules. (**b**) Correlation heatmap between liver and muscle modules. Each square shows the correlation coefficient between the module eigengenes (MEs) of the indicated modules from the two tissues. Module names are shown along the axes. Left panels present Pearson correlation coefficients; right panels present Spearman correlation coefficients. The color scale indicates correlation strength and direction (blue = positive, red = negative), with intensity proportional to the absolute value. Numbers indicate correlation coefficients, and asterisks denote significance (* *P* < 0.05; ** *P* < 0.01; *** *P* < 0.001).
